# Thymosin Beta 4 Protects Mice from Monocrotaline-Induced Pulmonary Hypertension and Right Ventricular Hypertrophy

**DOI:** 10.1371/journal.pone.0110598

**Published:** 2014-11-20

**Authors:** Chuanyu Wei, Il-Kwon Kim, Li Li, Liling Wu, Sudhiranjan Gupta

**Affiliations:** 1 Division of Molecular Cardiology, Department of Medicine, College of Medicine, Texas A & M Health Science Center and Scott & White, Central Texas Veterans Health Care System, Temple, Texas, United States of America; 2 Department of Physiology and Pathophysiology, Peking University Health Science Center, Beijing, China; Rutgers New Jersey Medical School, United States of America

## Abstract

Pulmonary hypertension (PH) is a progressive vascular disease of pulmonary arteries that impedes ejection of blood by the right ventricle. As a result there is an increase in pulmonary vascular resistance and pulmonary arterial pressure causing right ventricular hypertrophy (RVH) and RV failure. The pathology of PAH involves vascular cell remodeling including pulmonary arterial endothelial cell (PAEC) dysfunction and pulmonary arterial smooth muscle cell (PASMC) proliferation. Current therapies are limited to reverse the vascular remodeling. Investigating a key molecule is required for development of new therapeutic intervention. Thymosin beta-4 (Tβ4) is a ubiquitous G-actin sequestering protein with diverse biological function and promotes wound healing and modulates inflammatory responses. However, it remains unknown whether Tβ4 has any protective role in PH. The purpose of this study is to evaluate the whether Tβ4 can be used as a vascular-protective agent. In monocrotaline (MCT)-induced PH mouse model, we showed that mice treated with Tβ4 significantly attenuated the systolic pressure and RVH, compared to the MCT treated mice. Our data revealed for the first time that Tβ4 selectively targets Notch3-Col 3A-CTGF gene axis in preventing MCT-induced PH and RVH. Our study may provide pre-clinical evidence for Tβ4 and may consider as vasculo-protective agent for the treatment of PH induced RVH.

## Introduction

Thymosin β4 (Tβ4), a 43-amino acid actin-binding protein encoded by gene *Tmsb4x* on the X chromosome in mouse, displays abundant protective effects on diverse pathological conditions [1]–[3]. This includes promoting the migration of endothelial cells [4]–[7], accelerating angiogenesis [8], [9], downregulating inflammatory response[10]–[12] and inhibiting apoptosis and oxidative damage [11], [13]–[15]. At cardiac remodeling setting, it has been reported that treatment with Tβ4 prior to myocardial infarction improved cardiac performance, abrogated scar formation, and enhanced cardiomyocytes survival [16]–[18]. These cardio-protective effects may be the ability of Tβ4 to stimulate the differentiation of new coronary vascular cells like vascular smooth muscle cells, thereby, improving cardiac capillary bed formation including coronary [7], [19], [20]. Together, these data indicate that stimulation with Tβ4 may have positive effects on vessel formation and may inhibit disease progression post cardiac injury. In regards to the role of Tβ4 in lung disease progression, De Santis M *et al.* showed the presence of higher concentration of Tβ4 in bronchoalveolar lavage fluid of scleroderma lung disease patients [21], indicating a protective role against lung tissue damage.

Pulmonary hypertension (PH) is a critical cardiopulmonary disorder marked by increases in pulmonary artery pressure and pulmonary vascular resistance that causes significant morbidity and mortality in the world [22]. PH is a vascular disease that obstructs the pulmonary arteries. The disease is characterized by a progressive pulmonary vasculopathy which leads to increased pulmonary arterial pressure (PAP), right ventricular hypertrophy (RVH), fibrosis and RV failure. The pathogenesis of PH is attributed to the collective effects of vascular remodeling including pulmonary arterial smooth muscle cell (PASMC) proliferation, medial hypertrophy and pulmonary arterial endothelial cell (PAEC) dysfunction resulting in lumen obliteration [23]. Current therapies are limited and fail to fully reverse vascular remodeling [24]. Identifying key molecule for the treatment of PH is required for the development of new targeted therapeutics.

Previously, we have shown that monocrotaline (MCT)-induced PH-mediated RVH was prevented by cardiac and lung specific inhibition of NF-κB [25], [26]. We identified the BMP-SMAD-Id-Notch signaling axis which contributes a critical role in MCT-induced PH and RVH [25], [26]. Recently, Tβ4 was shown to protect mice from bleomycin-induced lung damage [27], indicating a possible role of this G-actin sequestering peptide in lung disease. Thus, investigating the mechanism by which Tβ4 coordinates the cellular function is a key to understand the underlying molecular mechanism of PH-induced RVH and fibrosis. The molecular pathways including BMP-Id-Notch signaling are thought to contribute a pivotal role in the development of MCT-induced PH in rodent model. However, the role of Tβ4 in this setting is currently unknown. This study is, therefore, designed to test a novel concept that Tβ4 may be considered for the treatment of PH in MCT-induced mouse PH model. The rational of this study is to elucidate how Tβ4 modulates the BMP-Id-Notch signaling pathways in the event of PH.

## Material and Methods

Twelve-week-old male mice (∼25 g) were used for experiments. The studies were conducted with the approval of Institutional Animal Care and Use Committee at the Texas A&M Health Science Center and Scott &White Hospital.

### Induction of pulmonary hypertension (PH)

The MCT-induced PH mouse model was developed as described previously [24]. Briefly, wild-type (WT) mice received an intraperitoneal (*i.p*) injection of MCT (80 mg/kg body wt) every 20 days for 3 times. Age- and sex-matched WT with normal saline-injected mice of C57BL/6 background served as controls. A total of six mice were studied in each group. For Tβ4 treatment, animals received an *i.p* injection of Tβ4 (200 µg/200 µl PBS) prior to MCT treatment. The control group received 200 µl PBS. The *i.p.* injection was given every day for 3 days following MCT for a week and then twice a week until the mice were euthanized. The tissues were collected for experimental use. All mice were fed standard rodent chow and provided water ad libitum.

### Determination of RV pressure and RVH

The RV pressure was determined using *iWorks* System, pressure catheter (CATH-SCI-1200) as described previously [24]. Briefly, mice were anesthetized with 3.5% isoflurane and subsequently maintained on 1.5% isoflurane during the procedure to maintain the heart rate about 450 beats per minute. The catheter was inserted into the right jugular vein and was advanced into the RV and pulmonary artery. The RV pressure and hemodynamic measurements were recorded using iWorks Software (iWorx IX/228S Data Acquisition System with the Scisense Advantage pV control unit version 5.0). All mice were euthanized after the procedure. The hearts and lungs were excised, flushed with cold PBS to remove blood. Hearts were then weighed and RV portions were carefully removed and RVH was determined as described [25], [26]. The samples were then frozen in liquid nitrogen and stored at −80°C for isolation of RNA and proteins or stored in 10% buffered formalin for morphological examination or histological staining.

### Morphological examination

Lungs and RVs were fixed in 10% phosphate-buffered formalin, stained with hematoxylin and eosin (H&E) and Masson's trichrome, respectively; photographed using a DP-72 color camera.as described previously [25], [26]. The sections were examined by bright-field microscopy using an Olympus microscope (Olympus, Tokyo, Japan) and photographed using a DP-72 color camera.

### RNA extraction and quantitative real-time polymerase chain reaction (q RT-PCR)

RNA was extracted from the lung and RV tissue of WT, Tβ4, WT+MCT and MCT+ Tβ4 mice using an RNEasy kit (Qiagen, Valencia, CA, USA), following the manufacturer's instructions. The qRT-PCR was performed using gene-specific primers as described previously [25], [26].

### Western blot analysis

The lung and RV heart tissue were pulverized in liquid nitrogen and the total proteins were extracted using tissue extraction reagents (T-PER, Pierce, Rockford, IL). For in vitro analysis, the cells were lysed with RIPA lysis Buffer (Santa Cruz Biotechnologies, Santa Cruz, USA). The Western blotting and the subsequent quantification of each blot were performed as described previously [25], [26]. The primary antibodies for BMPR2, Notch3 and Id1 were from Santa Cruz Biotechnologies (Santa Cruz, CA). Collagen type III A (Col 3a) antibody was purchased from Rockland Immunochemicals (Gilbertsville, PA, USA) and antibodies for CTGF and GAPDH were from Cell Signaling Technologies, Danvers, MA, USA).

### Culture of Lung Microvascular Endothelial Cells (MVEC)

Lung MVECs were purchased from VEC Technologies (Rensselaer, NY, USA). Cells were cultured and passaged in MCDB-131 complete medium (VEC Technology, NY). Lung MVECs were serum-free for 24 h before stimulation with TGF-β1 and MCT (25 µmol), separately. No cellular toxicity was observed at 25 µmol of MCT in lung MVEC.

### Culture of Lung fibroblasts

Lung fibroblasts were prepared from WT mice lungs using collagen dispersion method as described previously [13]. Lung fibroblasts were serum-free for 24 h before stimulation with Ang II.

### Statistical analysis

All experiments were performed at least three times for each determination. Data are expressed as the means ± SE and were analyzed using one-way ANOVA and secondary analysis for significance with Newman-Keuls Multiple comparison test, using Prism 5.0 GraphPad software (GraphPad, San Diego, CA). *P*<0.05 was considered statistically significant.

## Results

### Effect of Tβ4 on RV pressure and RVH

**Figure 1:**
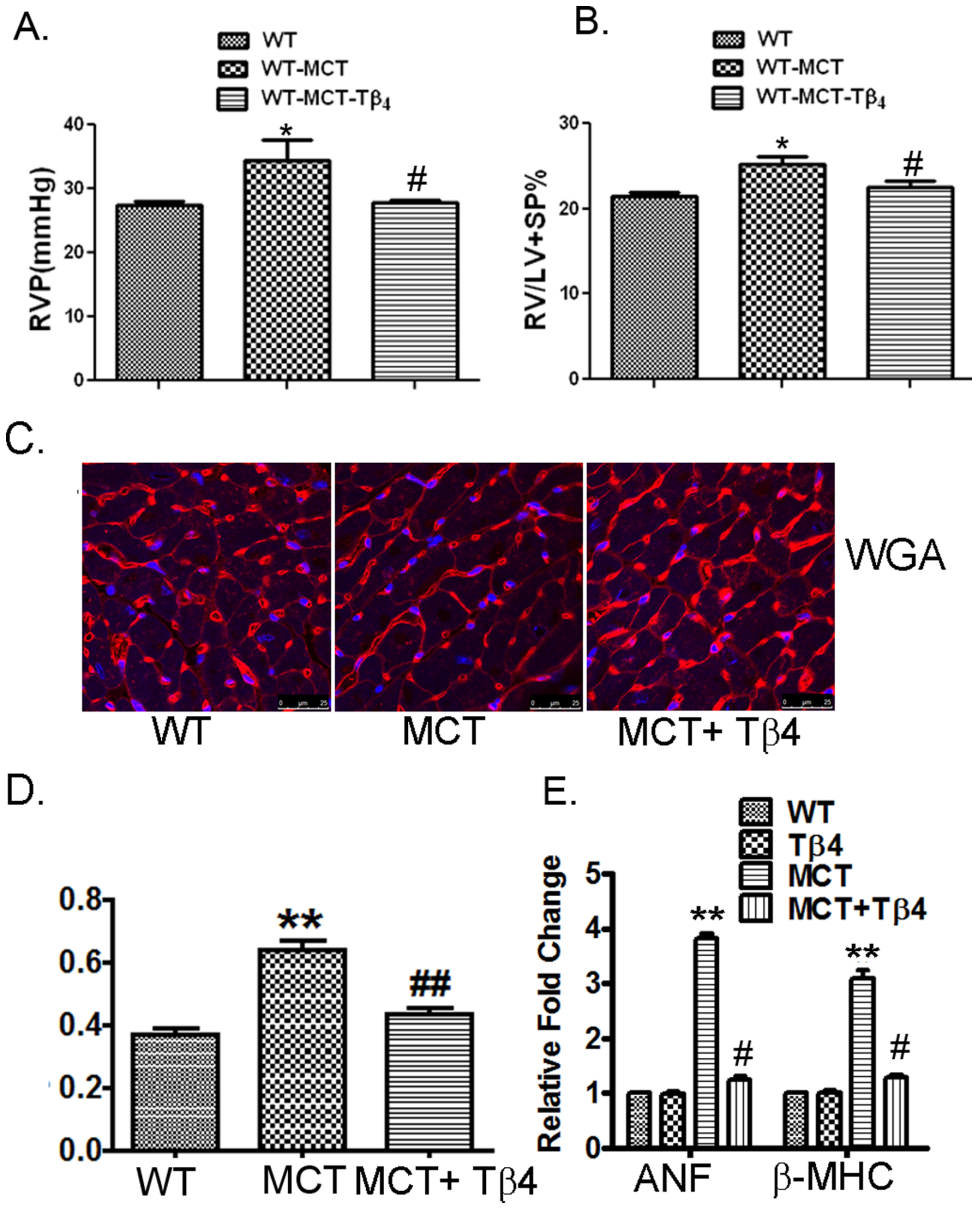
Tβ4 treatment reduced the right ventricle pressure and RVH induced by MCT treatment. (A) Representative of RV pressure measurement using *iWorks* pressure catheter showing the RV pressure in the WT, WT+MCT and MCT+ Tβ4 groups. Data are expressed as means ± SE from at least 3 independent mice. (B) The RV/LV+SP ratio (index for RVH) of the above group of mice. Data are expressed as means ± SE from at least 3 independent mice. (C) Representative images and averaged bar graphs of WGA staining for RV transverse section of WT, WT+MCT, and Tβ4+MCT mice. (D) The mRNA expression of ANF and β-MHC was determined by quantitative RT-PCR in WT, Tβ4, and WT + MCT, and MCT+ Tβ4 mice. *p<0.05 compared with the WT mice, **p<0.01 compared with the WT mice for ANF and β-MHC gene expression analysis and ^#^ *p*<0.05 compared with the WT+MCT mice.

The pathogenesis of MCT-induced PH was evaluated through RV pressure measurement. There was a significant increase in the RV pressure in the MCT-treated WT mice, compared with the untreated WT mice (37.51±1.843 *vs.* 27.53±1.062 mm Hg, *p*<0.05). Treatment with Tβ4 showed significant reduction in RV pressure (27.81±0.89*vs*. 37.51±1.843 mm Hg, *p*<0.05) in WT+MCT group, compared with the MCT-treated WT mice (Fig. 1A).

RVH was determined by a ratio of right ventricle (RV) weight to left ventricle (LV) plus inter ventricular septum (S) weight [RV/(LV+S)]. There was a significant increase in the RV/(LV+S) ratio in the MCT-treated WT mice (0.25±0.029, *p*<0.05), compared with the untreated WT (0.21±0.013) mice. Treatment with Tβ4 showed significant attenuation of RV/(LV+S) ratio (0.23±0.021) in the WT+MCT group, compared with the MCT-treated WT mice (*p*<0.05) (Fig. 1B).

To further evaluate the hypertrophic response induced by MCT in the RV, we determined myocyte cross-sectional area by wheat germ agglutinin (WGA) staining and analyzed hypertrophic marker genes. The representative images and averaged bar graphs from the WGA staining of hearts from WT, WT+MCT and MCT+ Tβ4 groups are shown in Fig. 1C. WGA staining showed a 1.72-fold (WT: 0.3719±0.017 *vs.* MCT: 0.6430±0.026, *p*<0.01) increase in cardiomyocytes cross-sectional area in the MCT-treated WT mice (Fig 1D). The MCT-induced right ventricular cardiomyocytes hypertrophy was significantly alleviated in MCT+ Tβ4 mice (MCT: 0.6430±0.026 *vs.* MCT+ Tβ4: 0.4372±0.020, *p*<0.01) (Fig. 1D).

Gene expression of ANF and β-MHC was increased to 3.8266±0.157-fold and 3.0833±0.262-fold (*p*<0.01) respectively, in MCT-treated WT mice, compared with the untreated WT mice. Significant reduction of ANF and β-MHC genes was observed in MCT+ Tβ4 mice (1.247 ±0.112-fold and 1.292±0.080-fold, *p*<0.05, respectively), compared with MCT-treated group (Fig. 1E). There were no significant changes observed in Tβ4-treated mice.

### Effect of Tβ4 on MCT-Induced Lung and RV Injury

**Figure 2:**
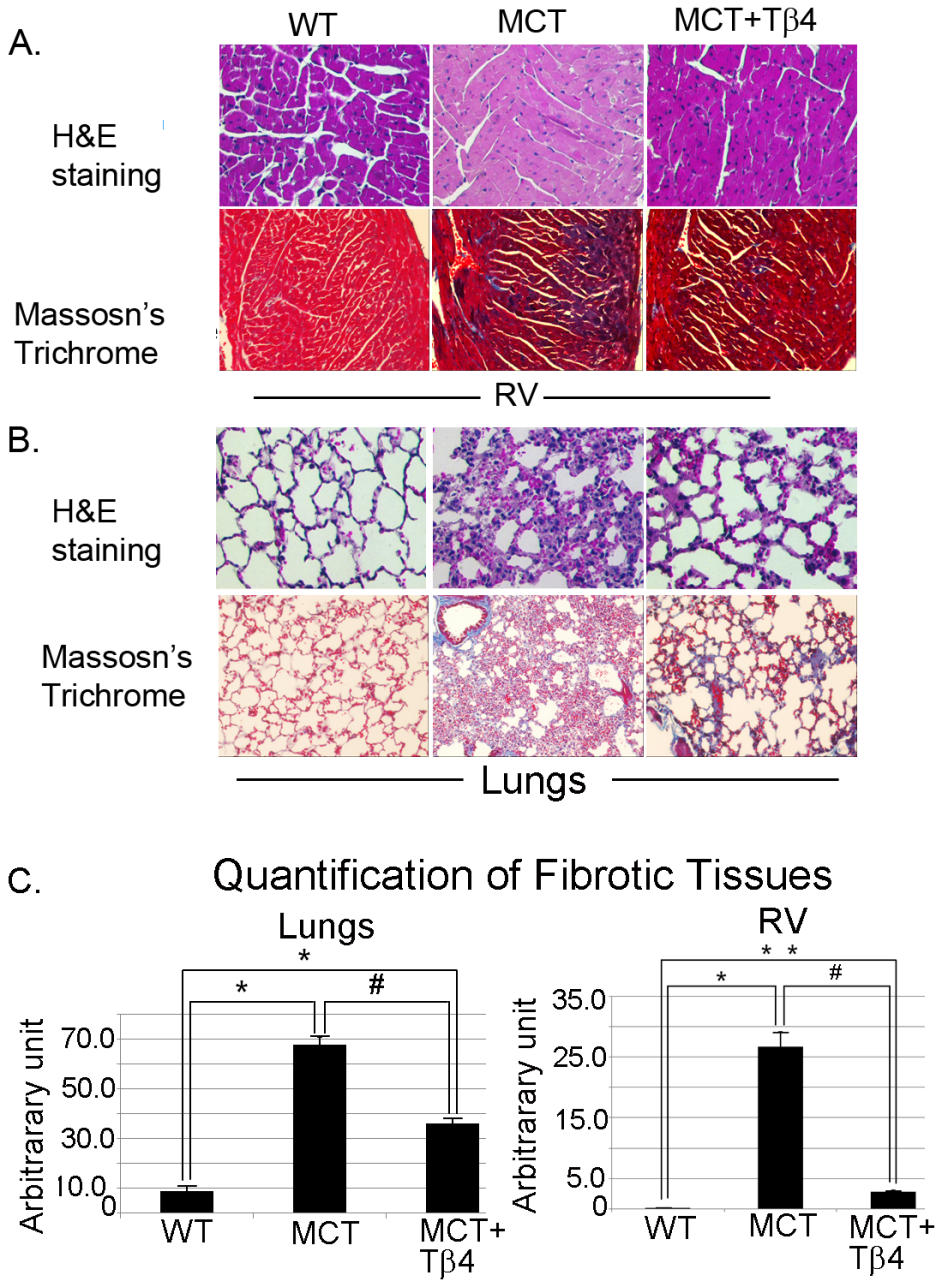
MCT-induced lung and RV injury in WT and Tβ4 treated mice. A. Representative images of RV section showing H& E staining (upper panel). The lower panel is showing Masson's trichrome staining. B. Representative of H&E staining images showing the MCT-induced lung injury in WT and Tβ4-treated mice (upper panel). The lower panel represents Masson's trichrome staining showing the MCT-induced collagen deposition in WT and Tβ4-treated mice. C. The graphs show the quantification of Fig A and B (lower panels) by densitometry. Data are expressed as means ± SE from 5 independent mice. For lung quantification, *p<0.01 compared to WT mice and # p<0.01 compared to WT+MCT group. For RV quantification, *p<0.05 compared to WT mice and #p<0.05 compared to WT+MCT mice. **p<0.01 compared to WT *vs.* MCT+ Tβ4 mice.

The histological alterations in lung and RV damages are demonstrated in Fig. 2. The upper panel of Fig. 2A demonstrated the H&E staining of RV section and the lower panel showed Masson's trichrome staining of WT, WT+MCT and MCT+ Tβ4 groups. Our data showed enhanced fibrosis in the RV section (0.38±0.002*vs.*26.75±2.348), compared to WT mice. The level of fibrosis was significantly reduced in MCT+ Tβ4 group (2.831±0.197) when compared with WT+MCT counterpart. MCT injection triggered intense infiltration of macrophages in the lungs of WT mice treated with MCT (Fig. 2B, upper panel) and the normal structure of alveoli was lost in many areas. Treatment with Tβ4 significantly reduced these maladaptive changes in the MCT-treated WT mice (Fig. 2B, upper panel). Masson's trichrome staining showed increased collagen deposition (8.75±2.21 *vs.* 67.75±3.30) in the pulmonary interstitium in the MCT-treated WT mice, compared to untreated WT mice (Fig. 2B, lower panel). The MCT-induced lung interstitial fibrosis was significantly alleviated in the MCT+ Tβ4 mice (36.0±2.16), compared to WT+MCT mice (Fig. 2B). The quantification of tissue fibrotic areas of lungs and RV were shown in Fig. 2C.

### Tβ4 treatment alters the expression and level of Notch3 and ICAM1 but not BMPR2 and Id1 in the lungs

**Figure 3:**
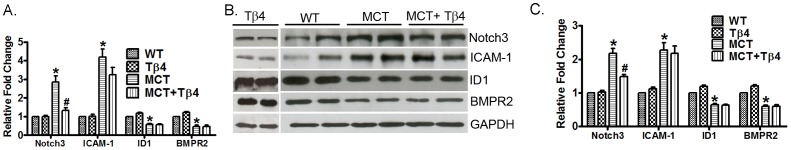
Effect of Tβ4 on MCT-induced changes of Notch3, ICAM1, Id1 and BMPR2 in mouse lung tissue. (A) Graph shows the mRNA expression of Notch3, ICAM1, Id1 and BMPR2 in WT, Tβ4, WT+MCT and MCT+ Tβ4 groups. The RT-PCR was performed using their specific probes. (B) Representative Western blots showing protein expression of Notch3, ICAM1, Id1 and BMPR2 in the above group of mice. GAPDH was used as an internal loading control. (C) Graph shows the relative fold change in the protein expression of Notch3, ICAM1, Id1 and BMPR2. Data are expressed as means ± SE from 5 independent mice.*p<0.05, compared with the WT mice. ^#^ *p*<0.05 compared with the WT-MCT mice.

To determine the protective effect of Tβ4 in the MCT-treated PAH mouse model, we evaluated the gene expression and protein level of BMPR2, Notch3 and ICAM1 in the lung tissue of WT, Tβ4, WT+MCT and MCT+ Tβ4 groups by real-time PCR and Western blot analysis. Real-time PCR analysis showed that MCT treatment increased Notch3 (2.847±0.5921, *p*<0.05) and ICAM1 (4.18±0.7742, *p*<0.05) mRNA expression; and attenuated Id1 (0.5972±0.06799, *p*<0.05) and BMPR2 (0.4686±0.1196, p<0.05) mRNA expression. The Tβ4 treatment showed significant reduction in Notch3 (1.34±0.2378, *p*<0.05) and ICAM1 (3.239±0.663) mRNA expression stimulated by MCT. We did not observe any changes or restoration of Id1 and BMPR2 expression (Fig. 3A). Western blot analysis showed that MCT treatment increased Notch3 (2.179±0.2614,*p*<0.05) and ICAM1 (2.272±0.3952, *p*<0.05) levels and decreased the Id1 (0.648±0.0703,*p*<0.05) and BMPR2 (0.5986±0.0646,*p*<0.05) levels in the mouse lung tissue (Fig. 3B and 3C). The Tβ4 treatment showed reduction only in Notch3 (1.488±0.1086,*p*<0.05) protein level stimulated by MCT. However, there was a slight reduction in the ICAM1 level.

### Tβ4 treatment significantly reduces Col 3a and CTGF gene expression and protein level in the lungs

**Figure 4:**
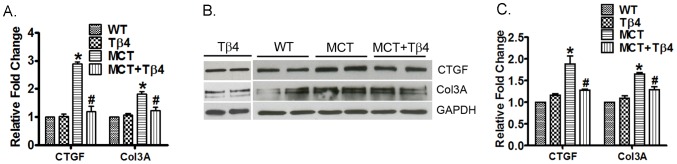
Effect of the Tβ4 treatment on CTGF and Col 3a expression in mouse lung tissue. (A) Graph shows the mRNA expression of CTGF and Col 3a in WT, Tβ4, WT+MCT and MCT+ Tβ4 groups. The RT-PCR was performed using their specific probes. (B) Representative Western blots showing protein expression of CTGF and Col 3a in WT, Tβ4, WT+MCT and MCT+ Tβ4 group. GAPDH was used as an internal loading control. (C) Graph shows the relative fold change in the protein expression of CTGF and Col 3a by densitometry. Data are expressed as means ± SE from 5 independent mice.*p<0.05, compared with the WT mice. ^#^ *p*<0.05 compared with the WT+MCT mice.

In order to determine whether Tβ4 has any influence on fibrosis, we evaluated the changes of Col 3a and CTGF in the lung tissues of WT, Tβ4, WT+MCT and MCT+Tβ4 groups by real-time PCR and Western blot analysis. WT mice treated with MCT showed significant up-regulation of CTGF (2.889±0.112, p<0.05) and Col 3a (1.812±0.165, p<0.05) mRNA in the mouse lung tissue. The mice treated with Tβ4 showed significant reduction in both CTGF (1.197±0.322, p<0.05) and Col 3a (1.238±0.206, p<0.05) mRNA expression (Fig. 4A). Western blot analysis showed that MCT treatment increased the CTGF (1.885±0.328, p<0.05) and Col 3a (1.656±0.058, p<0.05) protein level and Tβ4 treatment significantly attenuated CTGF (1.28±0.025, p<0.05) and Col 3a (1.286±0.132, p<0.05) level (Fig. 4B and 4C).

### Tβ4 treatment significantly inhibits the expression and level of Notch3 but not Id1 in the RV

**Figure 5:**
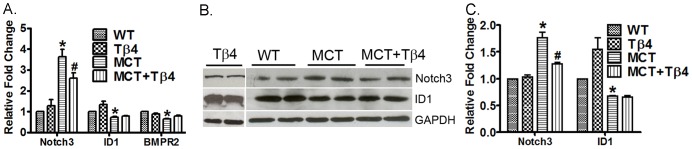
Effect of Tβ4 on Id1 and Notch3 expression in the mouse right ventricle. (A) Graph shows the mRNA expression of Id1 and Notch3 in the right ventricles of WT, Tβ4 WT+MCT and MCT+ Tβ4 groups. The RT-PCR was performed using their specific probes. (B) Representative Western blots showing protein expression of Id1and Notch3 in the above group of mice. GAPDH was used as an internal loading control. (C) Graph shows the relative fold change in the protein expression of Id1 and Notch3 by densitometry. Data are expressed as means ± SE from 5 independent mice.*P<0.05, compared with the WT mice. ^#^ *p*<0.05 compared with the WT+MCT mice.

The pathology of PH triggers the development of RVH and, we then determine the efficacy of Tβ4 on RVH in MCT-treated WT mouse. We further examined the expression of Notch3 and Id1 expression in the right ventricle of WT, Tβ4, WT+MCT and MCT+ Tβ4 groups by real-time PCR and Western blot analysis. Real-time PCR analysis showed a significant increment of Notch3 mRNA (3.631±0.613, p<0.05) and reduction in Id1 (0.731±0.076, p<0.05) and BMPR2 (0.6482±0.031, p<0.05) mRNA expression in right ventricles of MCT-treated WT mice. The Tβ4 treatment showed significant reduction in the Notch3 (2.603±0.445,p<0.05) mRNA only (Fig. 5A).Western blot analysis showed that MCT treatment up-regulated the Notch3 (1.765±0.189, p<0.05) level and down-regulated the Id1 protein level (0.676±0.013, p<0.05), compared to the MCT-treated mice. Importantly, the Tβ4 treated group showed significant reduction in Notch3 (1.276±0.606, p<0.05) protein level (Fig. 5B and 5C). We did not observe any significant changes in Id1 level in Tβ4-treated group.

### Tβ4 treatment significantly reduces Col 3a and CTGF expression and level in the RV

**Figure 6:**
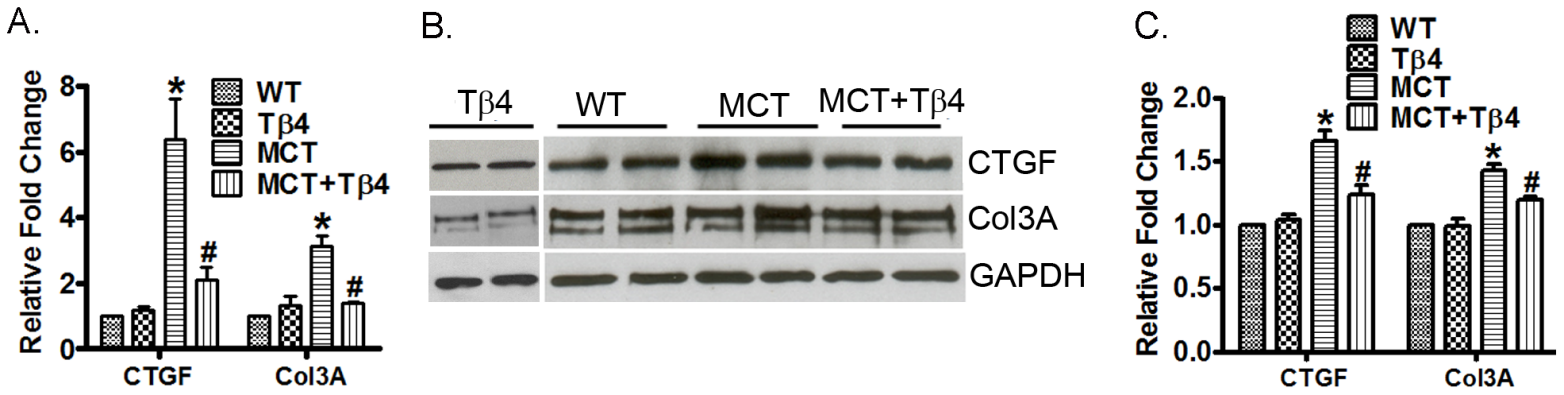
Effect of the Tβ4 treatment on CTGF and Col 3a level in the right ventricle. (A) Graph shows the mRNA expression of CTGF and Col 3a in the right ventricles of WT, Tβ4, WT+ MCT and MCT+ Tβ4 groups. The RT-PCR was performed using their specific probes. (B) Representative Western blots showing protein expression of CTGF and Col 3a in the above group of mice. GAPDH was used as an internal loading control. (C) Graph shows the relative fold change in the protein expression of CTGF and Col 3a by densitometry. Data are expressed as means ± SE from 5 independent mice.*p<0.05, compared with the WT mice. ^#^ *p*<0.05 compared with the WT-MCT mice.

In order to examine whether MCT-induced fibrosis is attenuated in the RV, we evaluated expression of CTGF and Col 3a in the WT, WT+MCT and MCT+ Tβ4 groups by real-time PCR and Western blot analysis. Real-time PCR analysis showed that MCT treatment increased CTGF (6.387±2.091, *p*<0.05) and Col 3a (3.146±0.516, *p*<0.05) mRNA expression in the mouse right ventricle. The Tβ4 treatment reduced the CTGF (2.11±0.639, p<0.05) and Col 3a (1.386±0.054, p<0.05) mRNA expression by MCT in the mouse right ventricle (Fig. 6A).Western blot analysis showed that MCT treatment increased CTGF (1.659±0.145, p<0.05) and Col 3a (1.428±0.081, p<0.05) protein levels in the mouse right ventricle. The Tβ4 treatment reduced the CTGF (1.24±0.119, p<0.05) and Col 3a (1.194±0.045, p<0.05) protein levels by MCT in the mouse right ventricle (Fig. 6B and 6C).

### Tβ4 treatment reduces TGFβ1 and MCT-induced Notch3, ICAM1 expression in lung microvascular endothelial cells (MVEC)

**Figure 7:**
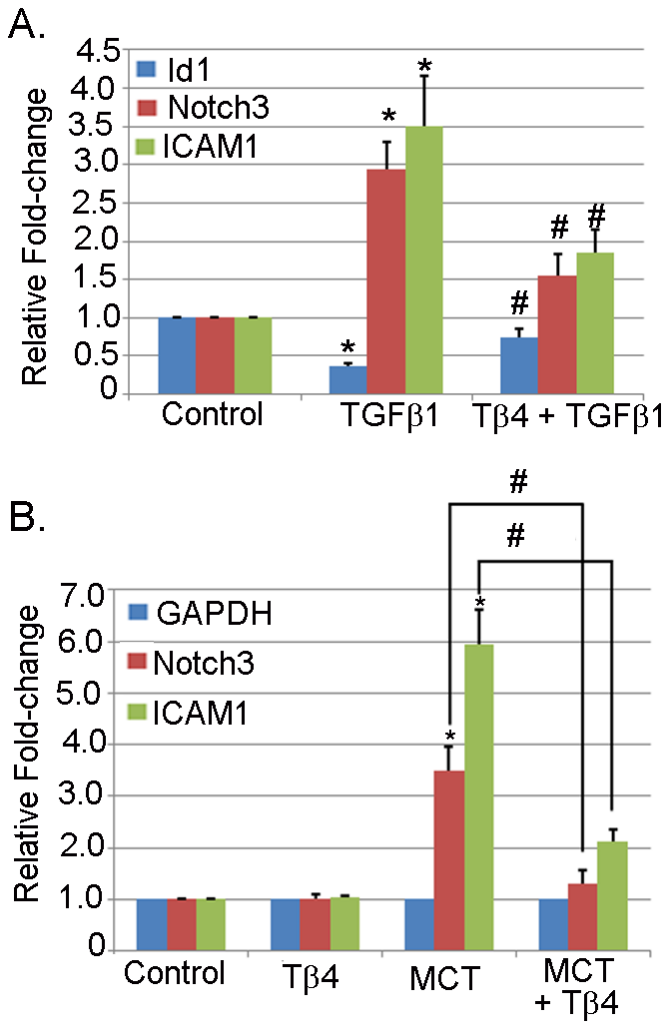
Effect of Tβ4 on Id1, Notch3 and ICAM1 expression in the lung MVEC. Lung MVEC was stimulated with TGFβ1 in the presence or absence of Tβ4. (A) The mRNA expression of Id1, Notch3 and ICAM1 were determined by qRT-PCR using their specific probes. The data presented are mean ± SE. *p<0.05 *vs*. control, ^#^ *p*<0.05 *vs*. TGFβ1 treatment (n = 3). (B) Lung MVEC was stimulated with MCT in the presence or absence of Tβ4. The mRNA expression of ICAM1 and Notch3 were measured by qRT-PCR using their specific probes. The data presented are mean ± SE. *p<0.05 *vs*. control, ^#^ *p*<0.05 *vs*. MCT treatment (n = 3).

To corroborate the *in vivo* studies, we performed our analysis using lung microvascular endothelial cells (MVEC) stimulated with TGFβ1. The TGFβ1 is a potent stimulus used in EC remodeling. To examine the efficacy of Tβ4 in vascular cell remodeling, we pretreated the cells with Tβ4 followed by the stimulation with TGFβ1. Our data showed that TGFβ1 significantly upregulated the Notch3 and ICAM1 (2.937±0.3546 and 3.50±0.6502, *p*<0.05, respectively) mRNA expression; and downregulated Id1 (0.37±0.03464) expression, compared to the unstimulated lung MVEC (Fig. 7A). The lung MVEC treated with Tβ4 showed significant attenuation of both Notch3 and ICAM1 (1.5466±0.2874 and 1.8566±0.3013, *p*<0.05, respectively); and partially restored the Id1 expression (0.735±0.1150, *p*<0.05). In separate experiments, we determined the expression of Notch3 and ICAM1 in MCT-stimulated lung MVEC. Our data showed that MCT treatment significantly upregulated both Notch3 and ICAM1 (3.493±0.4687 and 5.957±0.6705, *p*<0.05, respectively) mRNA expression, compared to the unstimulated lung MVEC (Fig. 7B). The lung MVEC treated with Tβ4 showed significant attenuation of both Notch3 and ICAM1 (1.3046±0.2633 and 2.1166±0.2417, *p*<0.05, respectively). It is also noted that lung MVEC treated with Tβ4 did not show any significant alteration of these genes.

### Tβ4 treatment attenuates Ang II-induced Col 3a and CTGF expressions in lung fibroblasts

**Figure 8:**
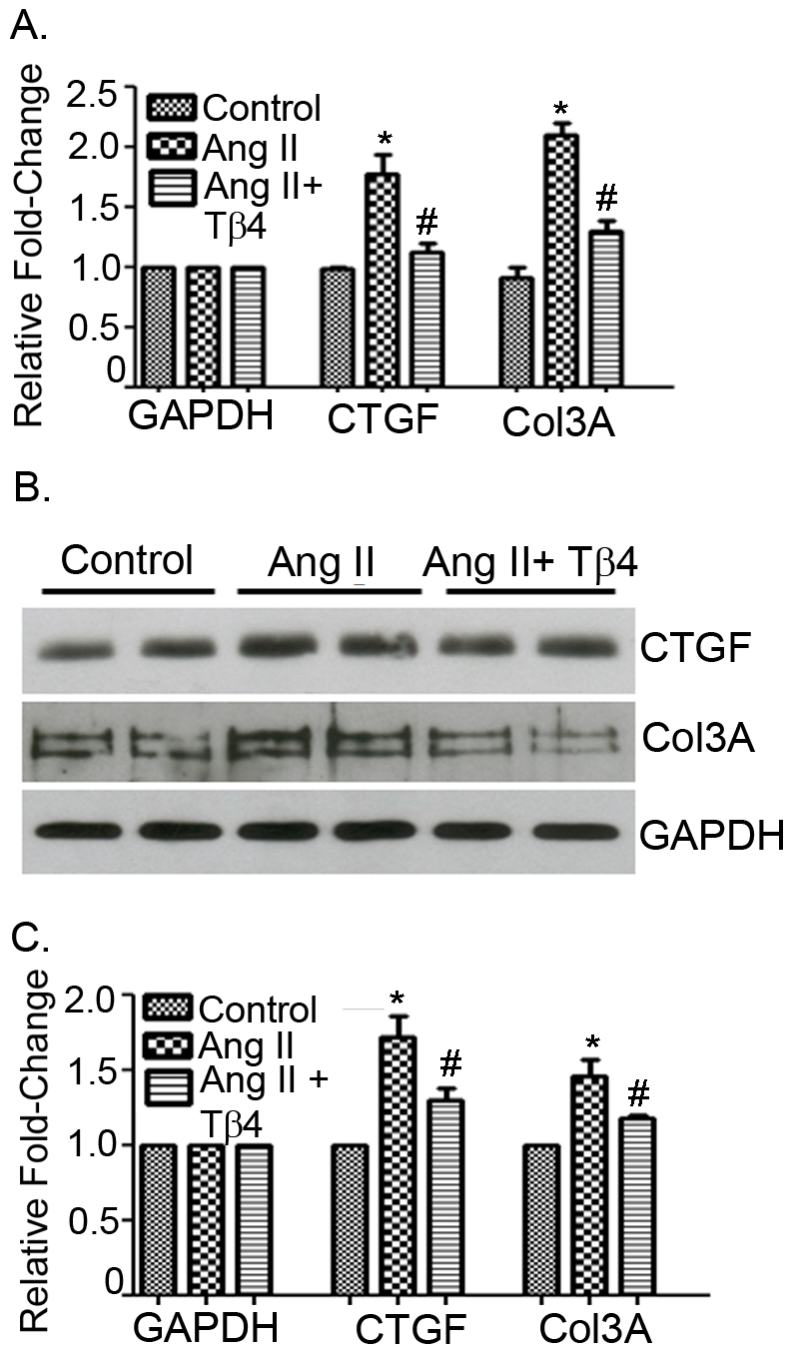
Tβ4 treatment attenuates CTGF and Col 3a in the lung fibroblast. Lung fibroblasts were stimulated with Ang II in the presence or absence of Tβ4. (A) The mRNA expression of CTGF and Col 3a were determined by qRT-PCR using their specific probes. (B) The protein level of CTGF and Col 3a were determined by Western blot using CTGF and Col 3a specific antibodies. (C) The quantification of Western blot is shown graphically. The data presented are mean ± SE. *p<0.05 *vs*. control, ^#^ *p*<0.05 *vs*. Ang II treatment (n = 3).

In order to determine the efficacy of Tβ4 treatment in fibrosis, we examined the expression and level of CTGF and Col 3a in lung fibroblasts stimulated with angiotensin II (Ang II). We used Ang II as a stimulus as it is well-accepted stimulus for pro-fibrotic cascade as shown us previously [13] The real-time PCR analysis showed that Ang II treatment increased CTGF (1.777±0.286, *p*<0.05) and Col 3a (2.099±0.168, *p*<0.05) mRNA expression in lung fibroblasts. Lung fibroblasts treated with Tβ4 showed significant attenuation of CTGF (1.132±0.115, *p*<0.05) and Col 3a (1.303±0.142, *p*<0.05) mRNA expressions, compared to Ang II stimulation (Fig. 8A). Western blot analyses showed significant enhancement of CTGF (1.721±0.145, *p*<0.05) and Col 3a (1.461±0.081, *p*<0.05) protein level in Ang II-stimulated lung fibroblasts. Lung fibroblasts prior treated with Tβ4 showed reduction in CTGF (1.304±0.119, *p*<0.05) and Col 3a (1.181±0.045, *p*<0.05) protein level, compared to Ang II stimulation (Fig. 8B and 8C). Lung fibroblasts treated with Tβ4 only did not show any significant alteration of Col 3a and CTGF genes (data not shown).

## Discussion

Our study demonstrates for the first time that Tβ4 may provide a protective role in MCT- induced PH and RVH mouse model. Our results indicate that Tβ4 selectively modulates the Notch3, Col 3a and CTGF genes and protects mice from the lung and heart damage induced by MCT administration. Moreover, by evaluating pertinent inflammatory markers, we provide evidence of significant attenuation of a signature of pro-inflammatory molecules in MCT-induced PH. We provide the following assertion by which Tβ4 offers protection in MCT- induced PH and RVH.

We showed that MCT treatment triggered the progression of PH as evidenced by increased RV pressure in WT mice and were associated with the development of RVH. Treatment of Tβ4 was able to attenuate the RV pressure and RVH indicated a possible protective effect in PH and RVH. The development of RVH in WT+ MCT mice was evidenced by an increase in cardiomyocytes cross-sectional area. The morphological changes were associated with alteration of BMPR2-Id-Notch-3 axis genes and upregulation of hypertrophic marker genes like ANF and β-MHC. WT+MCT mice treated with Tβ4 showed significant reduction in cardiomyocytes cross-sectional area, restoration of BMPR2-Id-Notch-3-axis genes and reduction of hypertrophy marker genes, indicating a great potential of therapeutic use in PH-induced RVH. Our data revealed inflammatory molecules which were triggered by MCT treatment were significantly reduced. Besides the anti-inflammatory effect, we demonstrated that Tβ4 was further conferred the restoration of lung damage initiated by MCT. This finding accorded with our previous finding in myocardial ischemia model [18]. Here, we showed that intraperitoneally administered Tβ4 substantially reduced alveolar damage, inflammatory cellular infiltrates, and interstitial edema and, alveolar exudates in MCT-treated mice. Tβ4 exerts protective effect against MCT-induced lung injury (or toxicity) possibly *via* scavenging mechanism. It is likely that MCT triggered the activation of inflammatory circuit and fibrotic effects by reactive oxygen species (ROS) generation [28]. It is reported that MCT increased pulmonary vascular permeability along with enhanced vasoconstrictor responsiveness in the lungs and alveolar capillary membrane permeability, inflammation and EC injury [29]–[31]. Our current study did not assess any ROS activity or anti-oxidative enzymes pattern in the course of PH development. However, it is more likely that Tβ4 acts as a ROS scavenger and may target the anti-oxidative enzymes which, is corroborated with our previous report [13]. Therefore, a future study on this aspect is warranted. Furthermore, our data provide a new observation of presence of significant fibrotic areas in the lungs of MCT-treated mice and were reduced in Tβ4-treated mice. The observation supports our previous report [18] along with the others findings in the setting of fibrotic event [27]. Together, our data underscore that treatment of Tβ4 reduces the lung damage, attenuates inflammatory responses and eventually restores the RV pressure.

In the line of target molecule analysis, our data indicated that Notch3-Col 3a-CTGF genes appear to be the prime target by Tβ4 in MCT-induced PH and RVH. Previously, we showed that BMPR2-Id-Notch3- axis genes played a critical role in MCT-induced PAH and RVH [25], [26]. In the current study, we observed that Tβ4 preferentially targeted Notch3; however, a slight restoration of BMPR2 was observed, but, not the Id molecules. Additionally, our data revealed more significant reduction of fibrotic genes like Col 3a and CTGF, the hallmark for fibrosis. The Notch system is highly conservative in evolution and plays an important role in cell proliferation, differentiation, and apoptosis [32]–[34]. Earlier report suggested that Notch3 was upregulated in PH patients and in hypoxia- or MCT-induced rodent model of PH and, mice with homozygous deletion of Notch3 did not develop pulmonary hypertension in response to hypoxic stimulation [35]. Our results supported this observation and suggested an important role of Notch signaling pathway in vascular remodeling in PH. However, it is of note that Notch signaling is complicated and we are still unaware of this pathway function in PH by different members of Notch; specifically, Notch1 and 4, as they are modulated differently depending on time, environment, and cell types. Therefore, further investigation is warranted to decipher the specific function of each Notch members in PH.

Our data further advocate a potential role of Tβ4 in fibrosis. We observed that there was an increase in the expression of pro-fibrotic genes like Col 3a and CTGF in MCT- induced PAH and RVH. Decreased expression of fibrotic genes is beneficial because it prevents fibrosis by reducing the extracellular matrix (ECM) deposition. Histological and molecular analysis revealed that significant ECM deposition (collagens) in the lung and RV sections was abrogated by Tβ4 treatment. This is the first report where we showed an anti-fibrotic property of Tβ4 in PH model. At molecular level, it remains unknown the underlying mechanism of anti-fibrotic nature of Tβ4 in PH model. However, it is reasonable to assume that Tβ4 may modulate the PI3K/Akt signaling to prevent fibrotic events as reported in liver fibrosis model [36], [37]. Another possibility is that Tβ4 may offer this protection through its anti-fibrotic tetra-peptide Ac-SDKP which is the first four amino-acid [38], [39]. It has been shown in rat renal dysfunction and fibrosis model that Ac-SDKP inhibits phosphorylation of smad2 followed by attenuation of fibronectin, interstitial collagen, and TGF-β1 level in the nephritic kidney [40]. Based on these reports, we may speculate that Tβ4 may modulate TGFβ signaling cascade in order to reduce fibrotic events. Future studies are currently underway to unfold the underlying molecular mechanism.

We also provide evidence that MCT-induced PH that leads to the development of RVH; was significantly attenuated by Tβ4. The morphological changes, gene expression patterns (Notch3-Col 3a-CTGF) and RV mass were significantly reduced by Tβ4 treatment.

Finally, to evaluate the effect of Tβ4 in cell culture setting (*an in vitro* analysis), we confirmed our data in TGFβ1-stimulated lung MVEC and Ang II-stimulated lung fibroblast cells, separately. We observed an upregulation of both Notch3 and ICAM1 and downregulation of Id1 in TGFβ1-stimulated lung MVEC and were significantly reduced in Tβ4 treatment except moderately restoration of Id1. In case of fibrotic process, our data further confirmed that Ang II-stimulated upregulation of both Col 3a and CTGF (the hallmark and markers for fibrosis) mRNA and proteins were significantly reduced in prior Tβ4 treatment in the lung fibroblasts. Together, our *in vitro* data suggest that upregulation of Notch3 may initiate the dysfunction of EC in the lungs which allows the penetration of infiltrating molecules at the site. These infiltrating molecules invade the barrier of neighboring cells like smooth muscle cells and activate the resident of adventitious fibroblasts. The activation of fibroblasts promotes the synthesis of ECM proteins and induces the fibrotic response.

In conclusion, our study demonstrates for the first time that Tβ4 protects the lung injury caused by MCT injection in mouse model. In parallel, our data provide evidence that Tβ4 may be considered as a new candidate for the treatment of PH and RVH. Recently, a randomized placebo controlled study was done in healthy volunteers to examine the dose and toxicity effect. The data showed no apparent toxicity effect, was well tolerated and is being considered to be used for acute myocardial infarction [41]. It is of note that Tβ4 has been considered for phase II clinical trial for acute myocardial infarction and stroke by RegeneRx pharmaceutical (http://www.regenerx.com). In light of this and our new insight of Tβ4's beneficial role in lung injury and fibrosis; our findings may offer therapeutic opportunity to treat PAH.

### Therapeutic implication

Our findings may provide a new therapeutic intervention for PH where pulmonary vascular remodeling occurs and are relevant in the clinical settings. Future studies are, therefore, warranted to examine the effect of Tβ4 under different experimentally induced PH models. We believe that Tβ4 is a possible therapeutic target as it has the ability to attenuate the expression of the critical genes in PH, like Notch3, collagen I and CTGF, thereby, alleviating the damage to the pulmonary bed in MCT-induced PH and RVH. These possibilities regarding the mechanisms whereby Tβ4 modulates the above molecules need to be further tested experimentally in future studies.

### Limitation of the Study

In this study, we evaluated the efficacy of Tβ4 in MCT induced PAH and RVH. However, the possibility of interaction between these genes and other molecular pathways cannot be ignored. Also other PH model like hypoxia or pulmonary artery banding in rodents needs to be further investigated under Tβ4 treatment.
