# Correction: Thymosin Beta 4 Protects Mice from Monocrotaline-Induced Pulmonary Hypertension and Right Ventricular Hypertrophy

**DOI:** 10.1371/journal.pone.0118428

**Published:** 2015-02-19

**Authors:** The PLOS ONE Staff

The following grant number is missing from the Funding section: 81311120472.

The complete, correct Funding section is: This study was partly supported by start-up funds from the Texas A & M Health Science Center, College of Medicine, by an American Heart Association- Grant-in-Aid (14GRNT 20490320), by Research Mentor Award by Scott & White Memorial Hospital and Texas A&M and National Natural Science Foundation of China to S. Gupta (SG) and Liling Wu (No. 81311120472). The funders had no role in study design, data collection and analysis, decision to publish, or preparation of the manuscript.
